# The technological invention of disease and the decline of autopsies

**DOI:** 10.1590/S1516-31802008000200001

**Published:** 2008-03-06

The medical literature has shown some concern about the steady decrease in the number of autopsies that are being performed, a well documented phenomenon in Europe,^1,2^ the United States^3,4^ and Latin America, including Brazil.^5–8^ This fall is evident even in countries where the procedure is mandatory, like Hungary.^9^

**Figure 1:**
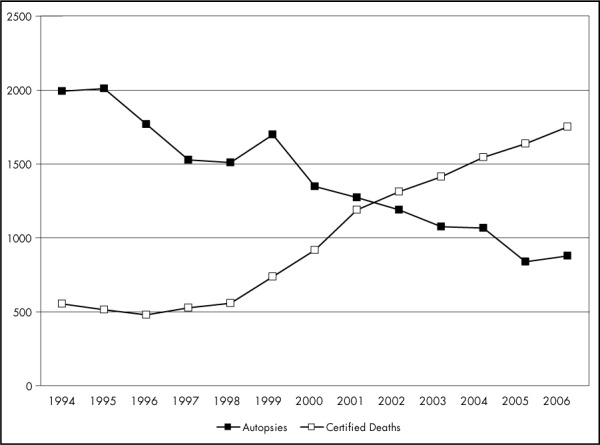
Rate of autopsied versus certified deaths at Hospital das Clínicas da Faculdade de Medicina da Universidade de São Paulo from 1994 to 2006.^10^

The frequency of autopsies performed at Hospital das Clínicas (HC), Faculdade de Medicina da Universidade de São Paulo (FMUSP), during the years 1996-2000 reached 75.6% of deaths (Figure 1).^10^ However, over the period 2001-2006 the proportion went down to 44.3%. These numbers are comparable with international statistics (Table 1).

**Table 1.** Autopsy rates according to country for two study periods

	Initial autopsy rate (period)	Subsequent autopsy rate (period)
Australia	21.0% (1992-93)	12.0% (2002-2003)
Brazil	75.6% (1996-2000)	44.3% (2001-2006)
France	15.4% (1988)	3.7% (1997)
Hungary	100% (1938-51)	68.9% (1992-2002)
Ireland	30.4% (1990)	18.4% (1999)
Jamaica	65.3% (1968)	39.3% (1997)
Sweden	81.0% (1984)	34.0% (1993)
United Kingdom	42.7% (1979)	15.3% (2001)
United States	26.7% (1967)	12.4% (1993)

Adapted from references.^1–9^

The reasons that explain these findings range from costs, families’ unwillingness and discrediting of the procedure among physicians who rely more on diagnostic techniques, to the fear of legal measures that may follow possible lack of matching between cause-of-death and treatments that had been administered, or simply a belief that the procedure is useless. Indeed, the studies cited above mostly point towards physicians’ attitudes as the main factor relating to this shortfall, either among clinicians or pathologists. Nonetheless, consistent and reliable cause-of-death data should assist in healthcare planning, and methodological ways of filling these gaps need to be found.^11^

From our point of view, this shortfall and all the reasons possibly cited as implicated are in fact consequences of a change in the concept of disease. The concept of disease can be considered to truly direct the art and science of medicine, thereby setting the course for the procedures to be followed, as well as the pathways for research.

Over the centuries, medical science has made use of different concepts of disease: some of them at the same time, as seen with the theory of germs in Pasteur's view and the debate between the ontological and physiological concepts of disease during the nineteenth century. For this reason, it would be reasonable to consider that medical science has used many different theoretical constructs as frameworks for understanding what human diseases could be. These frameworks have underpinned the social and cultural ambience of every epoch, thus empowering the discourse and knowledge of contemporary thinking.

Each of these frameworks has considered postmortem examinations differently. In fact, there would be no reason to study inanimate cadavers if disease is a disorder of the circulating humors (Hippocrates or Galen). Nonetheless, if disease is conceived of as a malfunction of parts or forms, like organs (Morgagni), tissues (Bichat) or cells (Virchow), “opening up a few corpses” becomes accepted, as theorized by Foucault.^12^ The latter attributed the new paradigm to Bichat, in which autopsies would play a special role, by guiding the medical focus to look for the space where diseases really act, thereby founding modern medicine.

But what could the contemporary framework now be? Why should autopsies now be dismissed instead of being used to find where diseases are located? What concept of disease continues for practicing and teaching medicine?

Hofmann has argued that the contemporary concept of disease is technologically constituted.^13^ This means that “technology provides the physiological, biochemical and morphological entities that are applied in defining diseases. It constitutes the formation of medical knowledge… and it strongly influences the explanatory models of disease and medical taxonomy.” The relationship between medicine and technology resembles the relationship between science and technology, but it is too complex to be discussed briefly. It suffices to state that medical science had not escaped the overwhelming power of technoscience imposed on the West.^14^

The technological invention of disease therefore represents a new paradigm. Accordingly, it would not be necessary to perform autopsies on bodies to correlate pathological features with any symptoms patients might have had, as in nineteenth century practice. Today, it would just be a case of checking images and lab results, since many, if not all, disease findings might be *defined* by those results. Autopsy may have been to Medicine what the particle accelerator has been to Physics: a field that allowed abstract thought to be tested in practice, in a way that provided a *link* between the subject and concept. Indeed, the theory is revisited from experimental results and goes further towards new understanding.

It is very interesting to note the heated debate going on in journals of Anatomy. There is a line of thought supporting the idea that surface anatomy and imaging can replace cadavers.^15–17^ There is even one medical school that already sponsors such teaching methods and seems to be proud of this.^18^ Indeed, this phenomenon has exactly the same explanation: as the image of disease is becoming dissociated from cadavers, the image of normality ought to be too. It makes much more sense to study the anatomy of a living being, either from the surface and surgical findings or from image representations.

The conclusion is that autopsies are a product of Cartesianism. The decline in the use of autopsies is the result of inadequacy of the modern conceptual framework for contemporary medical rationality. The present framework is now postmodern^19^ and comprises bits, images and other virtual elements. There is no place for corpses.
